# A single-cell transcriptomic atlas of complete insect nervous systems across multiple life stages

**DOI:** 10.1186/s13064-022-00164-6

**Published:** 2022-08-24

**Authors:** Marc Corrales, Benjamin T. Cocanougher, Andrea B. Kohn, Jason D. Wittenbach, Xi S. Long, Andrew Lemire, Albert Cardona, Robert H. Singer, Leonid L. Moroz, Marta Zlatic

**Affiliations:** 1 Howard Hughes Medical Institute Janelia Research Campus, Ashburn, VA USA; 2 Department of Physiology, Development, and Neuroscience, Cambridge University, Cambridge, UK; 3 Department of Zoology, Cambridge University, Cambridge, UK; 4 Department of Neuroscience and Whitney Laboratory for Marine Biosciences, University of Florida, Gainesville/St. Augustine, FL 32080 USA; 5 MRC Laboratory of Molecular Biology, Cambridge Biomedical Campus, Francis Crick Avenue, Cambridge, UK; 6 Department of Anatomy and Structural Biology, Albert Einstein College of Medicine, Bronx, NY USA

**Keywords:** Drosophila, Neuroscience, scRNA sequencing, Neurodevelopment

## Abstract

Molecular profiles of neurons influence neural development and function but bridging the gap between genes, circuits, and behavior has been very difficult. Here we used single cell RNAseq to generate a complete gene expression atlas of the *Drosophila* larval central nervous system composed of 131,077 single cells across three developmental stages (1 h, 24 h and 48 h after hatching). We identify 67 distinct cell clusters based on the patterns of gene expression. These include 31 functional mature larval neuron clusters, 1 ring gland cluster, 8 glial clusters, 6 neural precursor clusters, and 13 developing immature adult neuron clusters. Some clusters are present across all stages of larval development, while others are stage specific (such as developing adult neurons). We identify genes that are differentially expressed in each cluster, as well as genes that are differentially expressed at distinct stages of larval life. These differentially expressed genes provide promising candidates for regulating the function of specific neuronal and glial types in the larval nervous system, or the specification and differentiation of adult neurons. The cell transcriptome Atlas of the *Drosophila* larval nervous system is a valuable resource for developmental biology and systems neuroscience and provides a basis for elucidating how genes regulate neural development and function.

**Supplementary Information:**

The online version contains supplementary material available at 10.1186/s13064-022-00164-6.

## Background

Making sense of any complex system involves identifying constituent elements and understanding their individual functions and interactions. Neural circuit development and function is no exception. While recent advances in connectomics [1–9] and live imaging techniques [10–15] offer unprecedented information about neural connectivity and activity, these need to be combined with gene expression atlases to understand how genes regulate neural development and function. High-throughput single-cell RNA sequencing (scRNAseq) offers a way forward by providing a molecular-level identity for each cell via its transcriptomic profile (Taylor et al., 2021). Importantly, it is also scalable to populations of hundreds of thousands or millions of cells without incurring exorbitant costs. In the fruit fly, efforts are already well underway to produce connectivity [1–4, 7, 8], activity [11, 13–15], and behavior atlases [16, 17] of the nervous system. A major challenge is to combine genes, circuits, and behavior. Single-cell analyses have been performed in parts of the adult [18–20] or the larval nervous system at a single life stage [21, 22]. However, a comprehensive transcriptomic atlas of the complete central nervous system from multiple samples and across multiple stages of larval life was previously not available.

To this end, we developed a protocol to capture, sequence and transcriptionally classify the molecular cell types of the entire central nervous system of the *Drosophila* larva. We did this across 3 different life stages (1 h, 24 h and 48 h after hatching), providing a developmental profile of gene expression. Overall, our analysis reveals 67 distinct molecularly defined classes of cells in the larval nervous systems. We annotated these clusters based on the previously known markers. These included 31 distinct functional larval mature neuron clusters, 8 glial clusters, 6 neural precursor clusters and 13 developing immature adult neuron clusters. 5 clusters showed an abundance of mixed cell type markers and were excluded from further analysis. We identified genes enriched in each cell type both across distinct life stages and separately, at each life stage.

While scRNAseq provides detailed information about the transcriptional program deployed by a cell at the time of collection, a drawback of the technique is a loss of spatial information. In proof of principle validation experiments, we therefore used a recently developed RNA fluorescent in situ hybridization (RNA-FISH) protocol to resolve the anatomical location of a molecular cell type in the whole larval brain [23].

In summary, our gene expression Atlas for 62 distinct cell subtypes of the larval nervous system at 3 distinct developmental stages reveals a slew of candidate genes that could play a role in the development and function of these cell types. In a companion paper in the same issue, we explore in more detail the temporal patterns of gene expression across stages. Our gene expression Atlas presented in this study provides a valuable resource for the community and a basis for future investigation of molecular mechanisms underlying the development and function of the nervous system.

## Methods

### Contact for reagent and resource sharing

Further information and requests for resources and reagents should be directed to and will be fulfilled by the Lead Contact, Marta Zlatic (zlaticm@janelia.hhmi.org).

#### Fly stocks

*Drosophila* larvae were grown on standard fly food at 25 °C and kept in 12-h day/night light and dark cycle. Vials were timed by collecting eggs on a new food plate over the course of one hour.

Please see Supplementary Table 2 for *Drosophila* lines used in this study.

#### Single cell isolation

*Drosophila* larvae were dissected at 1 h, 24 h, 48 h, or 96 h after larval hatching (ALH). All dissections were performed in a cold adult hemolymph solution (AHS) with no calcium or magnesium at pH 7.4. Quality of single cell isolation was investigated by visual inspection with compound and confocal microscopy. Samples were placed on ice during waiting periods. Samples were isolated and run on the 10 × Chromium Single Cell 3’ immediately after cell dissociation.

First, the complete central nervous system (CNS) was dissected from every animal. The dissected nervous systems were kept in cold AHS on ice. For those samples where the brain and the ventral nerve cord (VNC) were sequenced separately, the separation of the brain from the VNC was performed using fine-tipped forceps and MicroTools (Cat #: 50–905-3315, Electron Microscopy Sciences). The time from digestion (the part of the protocol most likely to induce cell stress) to on the 10 × Genomic instrument was never longer than 30 min.

After separation of the brain from the VNC, the desired tissue was placed in 18 μL of AHS on ice. Once all samples were prepared, 2 μL of 10 × neutral protease (Cat #: LS02100, Worthington Biochemical Corp, Lakewood, NJ, USA) was added to a final volume of 20 μL. The intact brain tissue was digested for 5 min. The tissue was then transferred to a fresh drop of 20 μL of AHS.

Each sample was disaggregated with a clean, thinly pulled glass electrode until no tissue was visible under a dissection microscope. All debris (pieces of nerve and undigested tissue) were removed. Samples with fluorescent markers were observed under a fluorescence microscope to approximate cell density. The samples were then loaded onto the 10 × Chromium chip.

#### 10X Genomics

Single cell capture and library construction was performed using the 10 × Chromium microfluidic device and the Chromium Single Cell 3’ v2 Library and Gel Bead Kit (10 × Genomics, Pleasanton, CA). Manufacturer’s recommendations were followed for cell collection and library preparation. Samples were sequenced with an Illumina HiSeq following manufacturer’s instructions.

#### mRNA in situ hybridization

FISH probes were designed based on transcript sequences using the online Stellaris Designer and purchased from Biosearch Technologies. The probe is 18-22nt long with a 3’ end amine-modified nucleotide that allows directly couple to an NHS-ester dye according to the manufacturer’s instructions (Life Technologies). Dye-labeled probe was separated from the excess free dyes using the Qiagen Nucleotide Removal Columns. FISH protocol was described previously (Long et al,. 2017; [24]. The brains of 3rd instar larvae were dissected in 1xPBS and fixed in 2% paraformaldehyde diluted PBS at room temperature for 55 min. Brain tissues were washed in 0.5% PBT, dehydrated, and stored in 100% ethanol at 4 °C. After exposure to 5% acetic acid at 4 °C for 5 min, the tissues were fixed in 2% paraformaldehyde in 1xPBS for 55 min at 25 °C. The tissues were then washed in 1 × PBS with 1% of NaBH4 at 4 °C for 30 min. Following a 2 h incubation in prehybridization buffer (15% formamide, 2 × SSC, 0.1% Triton X-100) at 50 °C, the brains were introduced to hybridization buffer (10% formamide, 2 × SSC, 5 × Denhardt's solution, 1 mg/ml yeast tRNA, 100 μg/ml, salmon sperm DNA, 0.1% SDS) containing FISH probe at 50 °C for 10 h. and then at 37 °C for an additional 10 h. After a series of wash steps, the brains were dehydrated and cleared in xylenes.

#### Confocal and BB-SIM Imaging

For confocal imaging, the tissues were mounted in DPX. Image Z-stacks were collected using an LSM880 confocal microscope fitted with an LD LCI Plan-Apochromat 25x/0.8 oil or Plan-Apochromat 63x/1.4 oil objective after the tissue cured for 24 h. For single-molecule imaging, we use a previous described Bessel beam selective plane illumination microscope (BB-SIM). Detail construction of microscope and the imaging procedure was described previously [23]. Briefly, this BB-SIM is engineered to image in medium matched to the measured refractive index (RI) of xylene-cleared *Drosophila* tissue with axial resolution of 0.3 µm and lateral resolution of 0.2 µm. For BB-SIM imaging, the tissues were mounted on a 1.5 × 3 mm poly-lysine coated coverslip attached to a 30 mm glass rod. The imaging process requires the objectives and tissues immersed in the imaging medium consist with 90% 1,2-dichlorobenzene, 10% 1,2,4-trichlorobenzene with refractive index = 1.5525. Two orthogonally mounted excitation objectives are used to form Bessel beams, which are stepped to create an illumination sheet periodically striped along x or y, while a third objective (optical axis along the z direction) detects fluorescence. To employ structured illumination analysis, we collect multiple images with the illumination stripe pattern shifted to tile the plane in x, and repeat the process orthogonally to tile the plane in y. The sample is then moved in z, and the imaging repeated, and so on to image the 3D volume.

### Single cell bioinformatic analysis pipeline

#### Expression matrix generation

Cell by count matrices for each sample were obtained with Cell Ranger software (Version 1.3.1, 10 × Genomics, Pleasanton, CA, USA) and analyzed with the R package Seurat [25] in a reproducible environement generated by Guix with all necessary packages by running “guix environment -N –ad-hoc -m environement.scm”. The code and the environement description file can be accessed at https://github.com/histonemark/Brainseq_code.

Cell Ranger was used to perform sample demultiplexing, genome alignment, read quality filtering, and quantification. The output was a cell-by-features matrix of counts for each individually indexed and sequenced sample.

#### Quality control, individual sample processing and integration

In order to analyze the samples coming from different dissections and development age and remove batch effects coming from different sequencing runs we integrated the samples to a shared reduced dimensional space using the reciprocal PCA pipeline implemented in Seurat. Briefly, each sample was read as Seurat object and quality filtered retaining all cells with more than 200 genes detected and with a mitochondrial gene content below 20%. Each sample was individually lognormalized and its top 5000 variable genes selected. In order to find the matched expression states across samples 5000 features where used. Prior to integration each sample was individually scaled and its dimensionality reduced to the first 100 principal components. The anchors for integration were selected among the first 50 principal components for the 5000 features previously selected. Finally the reciprocal PCA integration was computed for the first 50 principal components.

#### Unsupervised clustering and annotation

After integration, all samples were analyzed together with the “standard Seurat workflow” for non conventional, non integrated samples: The expression of all genes was scaled, a principal component dimensionality reduction was computed and the first 50 components retained. Unsupervised cell classification was achieved with the Seurat FindNeighbors algorithm across the 50 principal components previously calculated. Clusters from this classification were obtained with the Seurat FindClusters algorithm on the 50 principal components and the resolution parameter fixed at 2. For cluster visualization purposes a two-dimensional reduction was calculated with UMAP on the same 50 principal components.

To annotate the identity of the discovered clusters we calculated their differential gene expression with the Seurat command FindAllMarkers changing the default parameters *logfc.threshold* to 0.1, *min.pct* to 0.1, *min.diff.pct* to 0.1, the *return.thresh* to 0.0501 and retained only positive enrichments changing *only.pos* to TRUE. Supplementary spreadsheet 2 contains the list of markers per cluster obtained. Cluster identity was ascertained by manual inspection of previously described cell-type markers as top hits for each particular cluster. A list of all the markers used and the literature references granting their use as cell type specific is summarized in Supplementary Spreadsheet 19.

#### Novel cell-type specific gene discovery

Dotplots detailing the expression makeup of particular cell types were generated in main figures for the top 60 cell-type enriched genes after filtering for genes with an average log2 fold change greater than 1 and only if present in more that 19% of the cells of the category tested. For visualization purposes the cell types of interest were always positioned in the bottom of the Y-axes. The dotplots containing all genes passing the aforementioned thresholds are included as Supplementary figures.

#### Temporal expression

To calculate differential gene expression per cluster and stage, we iteratively applied the Seurat function FindAllMarkers to each cluster after segregating its cells by stage (i.e. 1 h, 2 h or 48 h). The parameters were identical to the ones used in cluster annotation discussed above.

#### Differential expression among subclasses or superclasses

To identify gene expression differences between closely related cell types, we subset the classes and re-run the FindMarkers algorithm to increase the power and detect genes that differentiate the subclasses of interest. For example, for mature neuron markers, we created a new subset containing only mature neurons and aggregated same neuron types (Cholinergic, GABAergic etc.) under the same class.

Conversely all major cell-types (Mature neurons, Immature neurons, NPCs, Glia etc.) were aggregated before running the FindMarkers algorithm to facilitate the discovery of cell type markers at a lower level of granularity.

## Results

### Single cell transcriptomics atlas of the Drosophila larval central nervous system

**Fig. 1:**
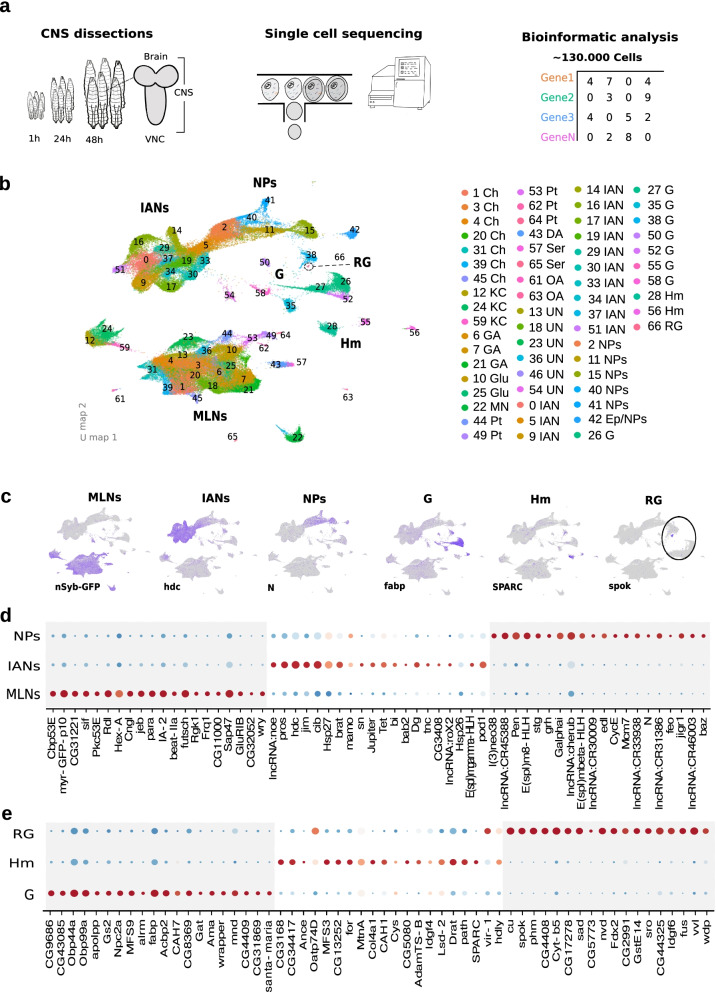
Generation of an integrated single-cell atlas of larval cell types across time and tissues. **a** *Experimental and analysis pipeline summary*. In order to generate an expression atlas across time and tissues, we dissected central nervous systems from larvae aged 1 h, 24 and 48 h post-hatching. Given the different neuron numbers present in the brain and ventral nerve cord (VNC), some dissections were exclusively brain or VNC. Refer to Supplementary table 1 for the concrete origin of each sample. Each dissection was treated as an idependent sample. We used 10XGenomics microfluidic technology for single-cell barcoded library generation followed by deep sequencing. We subsequently used Cell Ranger to generate cell by gene count matrices and the R package Seurat to integrate all samples to a common gene expression space allowing their joint analysis. **b** *All-stages, all-tissues integrated UMAP plot*. UMAP representation of the CNS cell type diversity discovered after reciprocal-PCA integration, dimensionality reduction and unsupervised clustering with Seurat. In this 2D representation each dot represents a cell and their distribution in space is a function of their similarity in gene expression profile. Each cluster is color and number coded as depicted in the accompanying legend. In order to characterize the cell-type identity resulting from the unsupervised clustering we ran differential gene expression in Seurat. Each cluster identity was annotated after inspection of the presence of previously described cell-type markers. MLNs: Mature Larval Neurons, Ch: Cholinergic Neurons, KC: Kenyon Cells, GA: Gabaergic Neurons, Glu: Glutamatergic Neurons, MN: Motorneurons, Pt: Peptidergic Neurons, DA: Dopaminergic Neurons, Ser: Serotoninergic Neurons, OA: Octopaminergic Neurons, UN: Unknown Neurons, IAN: Immature Adult Neurons, NPs: Neuroprecursors, Ep/NPs: Epithelia/ Neuroprecursor, G: Glia, Hm: Hemocytes, RG: Ring Gland. **c** *Feature plots for the marker distribution separating major CNS cell-types in UMAP space*. Mature neurons, i.e. larval functional neurons, express UAS-GFP under the pan-neuronal driver nSyb-GAL4. Immature neurons show high headcase (hdc) expression. Neuro-precursor cells are marked by Notch (N). The different glial classes express fatty acid binding protein (fabp), hemocytes are marked with Secreted protein, acidic, cysteine-rich (SPARC) while the ring gland can be differentiated by spookier (spook) expression. **d** Dotplots depicting the 20 topmost selectively enriched markers for Mature Larval Neurons, Immature Adult Neurons and Neuro-precursor cells. **e** Dotplots depicting the 20 topmost selectively enriched markers for Glia, Hemocytes and the ring gland. In each dotplot, the centered mean expression of a gene for each class is calculated and given a color ranging from blue (lowest expression) to red (highest expression), with white corresponding to 0. In this fashion different genes can be compared by their relative expression in the classes depicted irrespective of their absolute expression levels. The diameter of each dot is proportional to the number of cells expressing that gene in the class

**Fig. 2:**
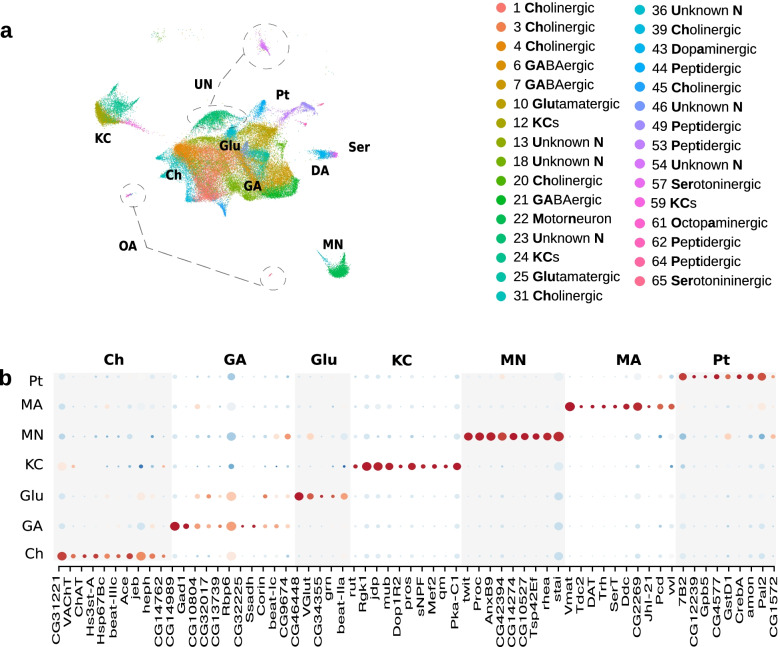
Mature neurons subclass analysis. **a** *UMAP plot of mature neuron classes.* A close-up look on mature neurons in UMAP space shows the subdivision of larval mature neurons in neurotransmitter (Cholinergic, Gabaergic, Glutamatergic, Peptidergic, Monoaminergic) and functional classes (KCs, Motorneurons). Glutamat.: Glutamatergic Neurons, Kcs: Kenyon Cells, Undetermin.: Undetermined Neurons, Dopamin.: Dopaminergic Neurons, Serotonin.: Serotoninergic Neurons, Octopamin.: Octopaminergic Neurons. **b** *Dotplot depicting the topmost selectively enriched markers for each mature neuron class*. Cholin.: Cholinergic Neurons, Kcs: Kenyon Cells, Gaba.: Gabaergic Neurons, Glut: Glutamatergic Neurons, Motor.: Motorneurons, Pepti.: Peptidergic Neurons and Monoam.: Monoaminergic Neurons

**Fig. 3:**
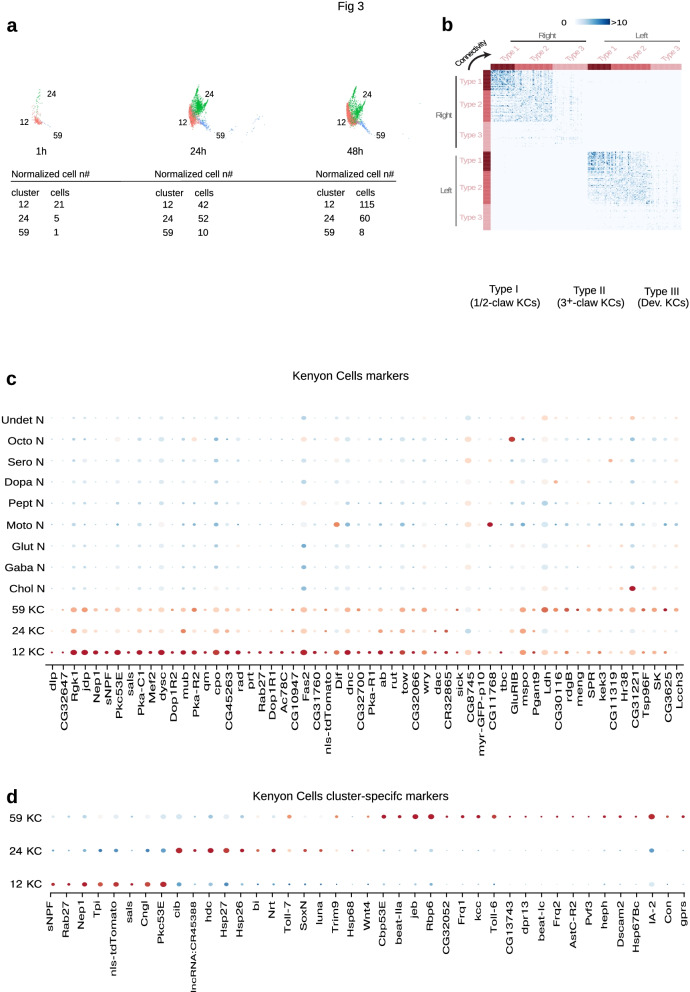
Connectome-consitent Kenyon Cell subtypes. Connectomic and developemental studies in Drosophila have established the temporal generation of morphologically distinct KCs during the larval stage. In agreement with this, the unsupervised clustering algorithm detected different KC clusters with different expression profiles. **a** *UMAP plot of the Kenyon Cells clusters separated by stage*. The cluster number corresponds to the numbering given in Fig. 1b. 1 h, 24 h, 48 h, refer to age in hours post hatching and prior to dissection. Each KC cluster comes from a different number of samples at each stage, the table shows the normalised the number of cells per stage at each stage. **b** Connectome KC cell types. Analysis of connectivity patterns among the larval KC population allowed their segregation in 3 morfological classes: 1/2-claw, 3 + -Claw and young. The matrix depicts the number of connections of all KC of the same and contralateral hemishpheres for each KC grouped by type. The intensity of blue of each matrix entry is proportional to the number of connections among 2 particular KCs. The bottom shows the volumetric reconstruction of the 3 classes of Kenyon Cells. **c** Dotplot depicting the topmost enriched KC markers compared to the other mature neuron clusters. KC: Kenyon Cells, Chol N: Cholinergic Neurons, Gaba N: Gabaergic Neurons, Glut N: Glutamatergic Neurons, Moto N: Motorneurons, Pept N: Peptidergic Neurons, Dopa N: Dopaminergic Neurons, Sero N: Serotoninergic Neurons, Octo N: Octopaminergic Neurons, Undet N: Undetermined Neurons. **d** Dotplot depicting the topmost enriched KC markers differentially expressed among the three KC clusters discovered in this study. KC: Kenyon Cells. Numbering as in Fig. 1b

**Fig. 4:**
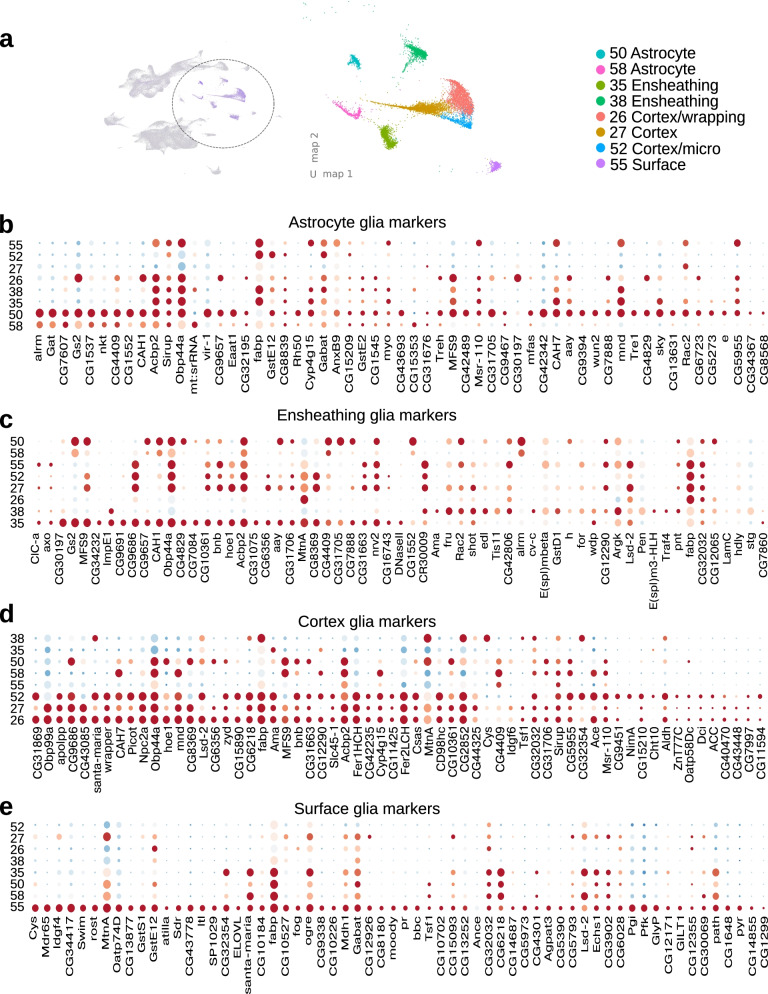
Glia subclass analysis. Glial cells are an heterogeneous population composed of morphologically and functionally distinct cells. Accordingly our atlas revealed clearly differentiated clusters occupying the same UMAP subspace owing to their common origin and expression profile. **a** *UMAP plot of the glial subclasses color and number coded.* The numbering corresponds to Fig. 1b. Inlet: Fabp positive feature plot indicating the origin of the glial clusters from atlas UMAP. **b** Atrocyte glial markers dotplot comparing their expression to the rest of glial subclasses. Bottom rows (cluster numbers 58, 50 correspond to Astrocyte glia). The numbering of the clusters is the same as in Fig. 1b. **c** Ensheathing glial markers dotplot comparing their expression to the rest of glial subclasses. Bottom rows (cluster numbers 35, 38 correspond to Ensheathing glia). The numbering of the clusters is the same as in Fig. 1b. **d** Cortex glial markers dotplot comparing their expression to the rest of glial subclasses. Bottom rows (cluster numbers 26, 27, 52 correspond to Cortex glia). The numbering of the clusters is the same as in Fig. 1b. **e** Surface glial markers dotplot comparing their expression to the rest of glial subclasses. Bottom rows (cluster number 55 corresponds to Surface glia). The numbering of the clusters is the same as in Fig. 1b

**Fig. 5:**
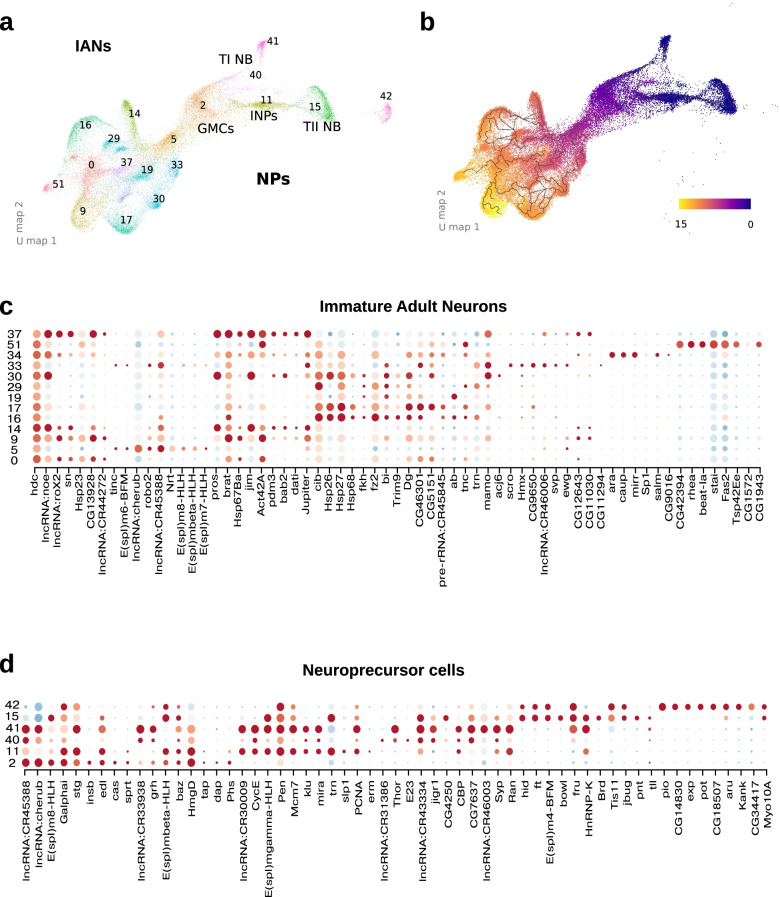
Immature neurons and neuro-precursor cells analysis. Thanks to our experimental design in which we gathered CNS at different stages of larval developpement, our atlas contains neurons at different stages along the differention axis neuroblast-functional neuron. **a** *UMAP plot of the differentiating neurons*. Neuroblasts and immature neurons at different stages of developpement cocluster sequentially along the first UMAP axis. The immature clusters occupy the lefmost part of the UMAP plot and are numbered as in Fig. 1b. Neuroblasts occupy the rightmost space and are numbered as in Fig. 1B and named after the presence of previously described markers. GMCs: Ganglion mother cells, INPs: Intermediate neural progenitors, TI NB: Type-1 neuroblasts, TII NB: Type-2 neuroblasts. **b** *Pseudotime trajectory analysis of the developping neurons*. To confirm the sequential co-clustering along the first UMAP axis we used the R package monocle3, to learn a pseudo-time trajectory in our developping neuron population. The trajectory was anchored at the cells with maximal Cyclin E (CycE) expression, and it’s depicted as a solid line starting at the INPs cluster. Cells are colored according to their pseudo-time value along the trajectory (see legend). Young cells (in pseudotime) coincide with neuroblast clusters while the oldest coincide with immature neuron clusters. **c** Immature neurons dotplot depicting the topmost enriched markers of each cluster. The numbering is the same as in Fig. 1b. **d** Neuroprecursor cells dotplot depicting the topmost enriched markers of each cluster. The numbering is the same as in Fig. 1b

**Fig. 6:**
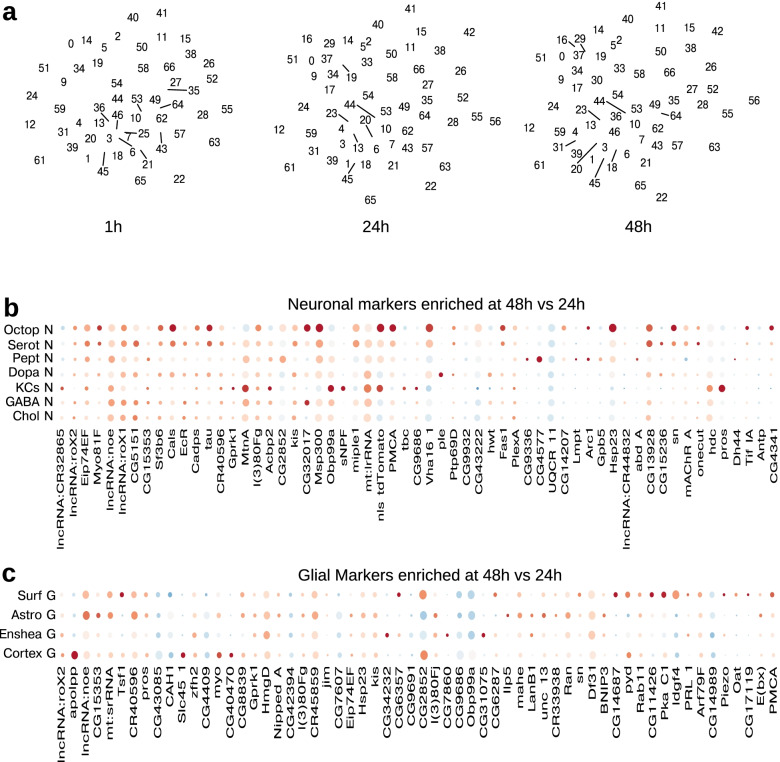
Temporal changes in marker composition. The Seurat algorithm of reciprocal-PCA allows to find matched populations of cells across our differently aged samples enabling the differential gene expression analysis of a given cell type at 1, 24 and 48 h age. **a** Same UMAP plot as in Fig. 1b, but separated per stage, showing the matched clusters when present. The numbering of the clusters is the same as in Fig. 1b. 1 h, 24 h, 48 h, refer to age in hours post hatching and prior to dissection. **b** Dotplot depicting the topmost temporally varible genes for mature neurons. Chol N: Cholinergic neurons, GABA N: Gabaergic neurons, KCs: Kenyon cell neurons, Dopa N: Dopaminergic neurons, Pept N: Peptidergic neurons, Serot N: Serotoninergic neurons, Octop N: Octopaminergic neurons. **c** Dotplot depicting the topmost temporally varible genes for glial subclasses. Cortex G: Cortex glia, Enshea G: Ensheathing glia, Astro G: Astrocyte glia, Surf G: Surface glia

To build a complete transcriptomic Atlas of the larval central nervous system (CNS) we captured cells at 3 distinct time points in development (1 h., 24 h., 48 h. after larval hatching) and for three kinds of nervous system dissections (full CNS, brain only and ventral nerve cord (VNC) only, Figs. 1, 2, 3, 4, 5 and 6, Supplementary Fig. 1 and Supplementary Spreadsheets 1, 2, 3, 4, 5, 6, 7, 8, 9, 10, 11, 12, 13, 14, 15, 16, 17, 18, 19, 20 and 21). We dissected out either the entire CNS, or just the brain or the VNC manually, dissociated the cells and sequenced their mRNA. In total we sequenced 131,077 cells that passed quality control. (See Supplementary Spreadsheet 1 for breakdown of samples for specific time points and tissues).

To enable the direct comparison of cellular states across stages and dissections we integrated all samples to a common reduced dimensional space with the reciprocal PCA algorithm implemented in the R package Seurat [26]. After integration, the joint analysis followed the standard pipeline implemented in Seurat: quality control, read-depth normalization, expression scaling, highly variable feature selection, dimensionality reduction and clustering based on gene expression similarity followed by a 2-dimensional representation with the Uniform Manifold Approximation and Projection algorithm (UMAP). (Fig. 1b) [26]. We could therefore analyze the same clusters across all stages and regions, either pooled (Figs. 1, 2, 3, 4 and 5, Supplementary Fig. 1 and Supplementary Spreadsheet 2), or separately for each stage (Fig. 6 and Supplementary Spreadsheet 3) and tissue (Supplementary Fig. 1, 2 and 3 and Supplementary Spreadsheets 4, 20 and 21).

Clustering of all cells revealed 67 different clusters (Fig. 1b). For each cluster, we identified all genes that were significantly differentially expressed in that cluster, relative to all other clusters (Supplementary Spreadsheets 2 and 6). We were able to identify and annotate 62 clusters based on their differential expression of previously known markers (Fig. 1b). The clusters fell into several very separate broad groups on the UMAP plot (Fig. 1b). One group comprised clusters of mature larval neurons; one comprised neural precursor cells (NPs) and developing adult immature neurons (IANs); one comprised glial cells (G) and ring gland cells (RG); and one comprised hemocytes (Hm, Fig. 1b-c). We also identified genes that were differentially expressed between these major groups (Fig. 1d-e).

The majority of neurons that participate in functional larval circuits and mediate larval behaviour are born from neural precursor cells (NPs) during embryonic development [27]. For a few of the larval neuronal cell types, such as the Kenyon cells (KCs) of the mushroom body (MB), new neurons are added during larval life [1],Pauls et al., 2010). We identified the functional mature larval neuron clusters (MLNs) based on their expression of classical neuronal markers, such as the sodium channel (*para*), and the component of neurotransmitter vesicle release machinery (*n-synaptobrevin*, *nSyb*, Fig. 1c-d, Supplementary Fig. 4 and Supplementary Spreadsheet 2) [28, 29]. In case some of these neuronal markers are expressed at very low levels, we had also expressed GFP under the control of the n-Syb promoter (nsyb-GAL4, UAS-GFP) in all our samples since the GAL4/UAS system [30, 31] amplifies expression (Fig. 1c-d and Supplementary Spreadsheets 6, 7, 8, 9, 10, 11 and 12, see Supplementary Table for fly stocks). We reasoned that the presence of the GFP transcript would confirm the identification of differentiated neuron clusters. Indeed, GFP was highly differentially expressed in 29 clusters. 27 of these clusters also expressed *para*. We labeled all 31 clusters that differentially expressed GFP (under nsyb promoter) and/or *para*, as clusters of functional mature larval neurons (MLNs, Fig. 1b-d, Supplementary Spreadsheet 5). Most of these clusters also differentially expressed endogenous *nSyb*, although at lower levels than GFP (which is amplified by the GAL4/UAS system). Our analysis identified further markers that were highly differentially expressed in mature larval neurons compared to other cell types in the nervous system (Supplementary Spreadsheets 2 and 5). In Fig. 1d, we show the top 20 genes with greatest differential expression (more than two-fold enrichment and expressed in more than 19% of the cells in those clusters) in mature larval neurons. These include a number of uncharacterized genes, such as *CG31221*, *CG11000*, and *CG32052*.

After hatching NPs remain quiescent for a while, but during larval life (mostly from early 2^nd^ instar and in some cases earlier than that) they start dividing again and producing new neurons that will become part of the adult nervous system [32, 33]). These developing immature adult neurons (IANs) are never integrated into functional larval circuits and do not contribute to larval behaviour, but they grow and elaborate parts of their projections, which they complete during pupation [34]. We identified 6 NP clusters and 13 immature adult neuron clusters (The NP clusters were identified based on their differential expression of *notch* (*N,* Fig. 1c-d and Supplementary Spreadsheets 2, 6 and 7, [35] and Pendulin [36]. Immature adult neurons were identified based on their highly differential expression of *headcase* (*hdc*) and/or *cibulot* (*cib*) [21], Fig. 1c-d and Supplementary Spreadsheets 2, 6 and 8). We identified a number of additional genes differentially expressed in immature adult neurons and NPs compared to fully differentiated neurons and other cell types (Supplementary Spreadsheets 2 and 6) and show top 20 genes that had the greatest enrichment in these classes in Fig. 1d.

Several types of non-neuronal clusters were also present in our dataset. 8 different clusters containing glia were identifiable based on their differential expression of *fabp* (Fig. 1c and 1e and Supplementary Spreadsheet 2) and of well-characterized markers for distinct types of glia, such as *wrapper* or *alrm* (Fig. 1e, 4a-e and Supplementary Spreadsheet 2 and 6, [37]. The classical cytogenetic marker of glia, reversed polarity (Repo) was detectable and specific for glia (Supplementary Fig. 5) but in accordance with previous larval scRNA-seq studies, exhibited sparse expression (Avalos, C.B. et al. 2019). A cluster of ring gland cells was identified based on differential expression of markers such as *spok* and *sad* (Fig. 1b-c, 1e and Supplementary Spreadsheet 2 and 6) [38]. 3 clusters containing hemocytes were identified based on expression of known markers, such as *SPARC* and *MetA* (Fig. 1b-c and e) [39, 40]. Genes differentially expressed in these classes of cells are also shown in Supplementary Spreadsheets 2 and 6 and Fig. 1e (top 20 with greatest enrichment).

Next, we analyzed in more detail individual clusters within each of the major categories above: mature larval neurons, glia, developing adult immature neurons and neural precursors.

### Identification of mature larval neuron clusters and their differentially expressed genes

We were able to identify most of the individual mature larval neuron clusters, based on their differential expression of specific neurotransmitters or neuropeptides and other previously reported markers (Figs. 1b and 2a-b, Supplementary Fig. 6, 7, 8, 9,10, 11 and 12 and Supplementary Spreadsheets 2, 5 and 6).

Six out of the 31 clusters did not differentially express any know neuron-type specific markers, so we called them unknown neurons (UN). This could be due to the relative shallowness of our sequencing resulting in a failure to detect genes that are expressed at low levels, or due to their expression of some unknown neurotransmitters.

Twelve clusters contained neurons that differentially expressed fast neurotransmitters (Fig. 2a-b, Supplementary Fig. 6, 7, and 8 and Supplementary Spreadsheet 2 and 5). Seven clusters expressed *VAChT* and therefore contained cholinergic neurons (Ch) [41], Supplementary Fig. 6). Three clusters expressed *VGlut* and therefore contained glutamatergic neurons (Glu) [42], Supplementary Fig. 7). One cluster had a very different pattern of gene expression from the other two and contained motor neurons (MNs) since it also differential expressed *twit* (Fig. 2b and Supplementary Spreadsheet 2, 5 and 6 [43]. 2 clusters expressed *Gad1* and were therefore annotated them as GABAergic (Supplementary Fig. 8 [44].

Three clusters were very segregated in the UMAP representation from other neuronal clusters and differentially expressed well known KC markers, such as *Dop1R2*, *rutabaga* and *prt* (Figs. 2a-b and 3a-d, Supplementary Spreadsheet 2, 5 and 6) [45, 46]. KCs are essential for associative memory formation and storage in insects [47] and are discussed in more detail in a separate section (Fig. 3a-d).

Monoaminergic neurons (MA) also play a key role in learning in the fly by providing the teaching signals for memory formation [48, 49],Selcho et al., 2014). We observed 5 different clusters of monoaminergic neurons with differential expression of *Vmat* (Fig. 2a-b, Supplementary Fig. 9, 10 and 11 and Supplementary Spreadsheets 2, 5 and 6 [50]: 2 octopaminergic (OA, differentially expressing *Tdc2, Tbh*) [51, 52], 2 serotonergic (Ser, differentially expressing *SerT* and *Trh*) and 1 dopaminergic (DA, differentially expressing *DAT* and *ple* (Supplementary Fig. 9, 10 and 11, Supplementary Spreadsheets 2 and 5) [53–55].

We further observed 4 distinct clusters of peptidergic neurons (Pt) that differentially expressed specific neuropeptides as well as *Phm* (Fig. 2a-b, Supplementary Fig. 12 and Supplementary Spreadsheets 2 and 5) [56]. 1 cluster (44) was very similar to the other 4 peptidergic clusters and also differentially expressed *Phm*, so we identified it as a putative peptidergic cluster, even though we could not detect differential expression of any known neuropeptides in this cluster.

In addition to these well-characterized neuron-type marker genes, we identified multiple genes whose expression was significantly enriched in each mature neuron cluster, compared to all other clusters (Supplementary Figs. 6, 7, 8, 9, 10, 11 and 12, Supplementary Spreadsheet 5). Many of these are CGs with unknown functions and provide interesting candidates for future studies.

Since neurons separated into distinct clusters based on their neurotransmitter expression, we wanted to identify further candidate genes that distinguish the major neurotransmitter classes. We therefore pooled clusters into several broader classes based on neurotransmitter expression (cholinergic, glutamatergic (non MN), GABAergic, monoaminergic, peptidergic, MN and KCs). KCs and MNs were so different from other larval neuron clusters in terms of gene expression and were very segregated in the UMAP location that we left them as separate classes and we did not pool them with other cholinergic or glutamatergic neurons [57, 58]. We then searched for genes differentially expressed between these major neuron classes (Fig. 2b and Supplementary Spreadsheet 5). We identified multiple genes whose expression was enriched more than two-fold in cholinergic, GABAergic, glutamatergic, peptidergic and monoaminergic neurons (the most differentially expressed genes for each subtype are shown in Fig. 2B). Some of these were differentially expressed only in a single neuron class, similar to the genes involved in neurotransmitter synthesis and transport. Thus, CG31221, CG46448, CG14274 were highly differentially expressed in cholinergic, glutamatergic and MN clusters respectively. CG14989 and CG32225 were selectively enriched in GABAergic neurons. Interestingly, CG14989 shares a promoter region with Gad1 (and the two are transcribed in different directions) with which it is tightly correlated in terms of expression, raising the possibility that their transcription could be co-regulated. C. elegans homolog of CG32225 (unc-46) is known to regulate GABAergic transmission [59].

Monoaminergic clusters were the only ones that differentially expressed CG2269. CG11317 was differentially expressed only in one cluster of serotonergic neurons. CG12239, CG14331, CG30053, CG13408, CG15537 and CG12541 were selectively differentially expressed in specific peptidergic clusters (Fig. 2B and Supplementary Spreadsheets 2 and 5).

We also wanted to identify all the genes whose expression distinguishes clusters of neurons that express the same neurotransmitter. Some genes were highly selectively enriched only in one or in very few specific clusters of the same neurotransmitter, compared to all other mature neuron clusters (Supplementary Fig. 13, 14, 15, 16, 17 and 18 and Supplementary Spreadsheets 7, 8, 9, 10, 11, 12, 13, 14, 15 and 16).

For example, the transmembrane Dpr interacting molecule, DIP-zeta, implicated in selective synaptogenesis was exclusively enriched in a single cholinergic cluster (cl. 1, Supplementary Fig. 13 and Supplementary Spreadsheet 9) [60]. A number of other DIPs or Dprs were also found to be differentially expressed in specific combinations of clusters (Supplementary Spreadsheet 9). The neuropeptide *space blanket* (*spab*) was highly differentially expressed in 2 of the cholinergic clusters (Supplementary Fig. 13 and Supplementary Spreadsheet 9) [61]. The zinc-finger transcription factors *disco* and *disco-r* were selectively enriched in the cholinergic cluster 39, and Oaz in cluster 45 and could potentially play a role in the specification of these cholinergic neuron types (Supplementary Fig. 13 and Supplementary Spreadsheet 9) [62–64].

The GATA family transcription factor *grain* (*grn*) was selectively differentially expressed in the glutamatergic cluster 25 (and the peptidergic cl. 44, Supplementary Fig. 14 and Supplementary Spreadsheets 2 and 11) [65].

In accordance with previous scRNAseq studies in adult [18, 19, 66] and larvae (Avalos, C.B. et al. 2019) we find neurons co-expressing two or three neurotransmitters (Supplementary Fig. 15 and Supplementary Spreadsheet 2). Understanding the significance of this phenomenon will require further functional studies.

Some of the markers that best distinguish individual clusters with the same neurotransmitter are also differentially expressed in other neurotransmitter classes. Such genes could nevertheless uniquely specify the identity of individual clusters in combination with a neurotransmitter gene. We therefore also identified genes that are differentially expressed among individual clusters of the same neurotransmitter phenotype (Supplementary Fig. 12, 13, 14, 15, 16, 17 and 18 and Supplementary Spreadsheets 9, 10, 11, 12, 13, 14 and 15).

These types of genes whose expression is enriched in a specific cluster compared to other clusters of the same neurotransmitter could be involved in the specification or function of specific neural types and provide interesting candidates for follow up studies.

### Transcriptomics reveals distinct larval KC types consistent with the connectome

We identified 3 distinct clusters of Kenyon cells, based on the expression of previously reported markers, such as *rut*, *DOP1R2, DOP1R1, prt*, and *mub* (Fig. 3a-c and Supplementary Spreadsheet 2) [42, 45–, 67–71]. In total, we found more than 90 differentially expressed genes with a more than twofold enrichment in the KCs clusters, including 17 CGs with previously unknown functions (Supplementary Spreadsheet 2). Some of these genes were fairly selectively expressed, either in all KC clusters, or in specific KC clusters, but not in many other mature neuron classes. For example, the actin-binding protein, *sals* was differentially expressed only in KC clusters (Fig. 3c and Supplementary Spreadsheets 2 and 15) [72]. The predicted integral membrane protein, *CG32647* was selectively differentially expressed only in KC clusters 12 and 59. The MADS-box family of transcription factor, Mef2, was highly differentially expressed in KCs and less so in one other cholinergic cluster (4). Mef2 has been shown to be expressed in adult MB and its orthologue has been implicated in learning and memory in the mouse [73]. The isoprenyl pyrophosphate synthase, *quemao* (*qm*), was highly selectively expressed in all KC clusters (and in one hemocyte cluster) [74]. The transcriptional cofactor *dachshund*, was expressed in KCs and in one peptidergic cluster [75]. These types of genes whose expression is highly enriched in KCs provide interesting candidates for potential involvement in learning and memory in the larval mushroom body.

Larvae were generally thought to have just one type of KCs, the gamma KCs. However, a recent connectome of the 1^st^ instar *Drosophila* larval MB revealed that KCs differ based on their connectivity [1]. Some KCs receive input just from a single olfactory projection neuron (PN) on a single dendritic claw,some receive input from two, three, four or more PNs on two, three, four or more dendritic claws. Furthermore, unlike other larval neurons, most of which are born during embryogenesis, KCs are continuously born and integrated into the functional larval mushroom body circuitry throughout larval life. There is a significant correlation between birth order and the number of claws, such that the single-claw KCs are born first, followed by two-claw KCs, then three-claw KCs etc. [1]. Additionally, the EM reconstruction revealed developing multi-claw KCs whose axons were still growing within the lobes. Clustering KCs based on their connectivity (not just PN input, but all their input and output) produced three distinct clusters, two clusters of mature KCs, corresponding to early-born KCs with fewer claws and later-born KCs with multiple claws, and a cluster of developing KCs whose axons were still growing (Fig. 3b). Two clusters of mature KCs differed in terms of other features besides their PN input patterns. Thus, the early-born KCs with fewer dendritic claws had many more connections with each other, than the later-born KCs with more dendritic claws (Fig. 3b).

Interestingly, our clustering of KCs based on gene expression also revealed three distinct clusters of KCs, similar to the number of clusters obtained based on connectivity. We analyzed these clusters separately, at each developmental stage: 1 h., 24 h. and 48 h. (Fig. 3a). One of these clusters (cl. 24) had many markers of developing neurons suggesting that it could contain the developing larval-born KCs. One cluster (cl. 59) had a relatively small number of cells (< 10) at all three stages (when normalized by sample number at each stage) and did not show a large increase in cell number between 24 and 48 h. We therefore speculate that this cluster could contain the embryonic-born single-claw KCs. Cluster 12 could contain the fully developed multi-claw KCs which have already been added into the larval circuitry. These assignments are tentative, and will have to be confirmed in the future by labeling each of the clusters with cluster-selective markers.

We therefore asked which genes differentiate individual KC clusters from each other (Fig. 3d and Supplementary Spreadsheet 15). We identified a number of genes that were more than two-fold enriched in a single KC cluster, compared to the others. The enhancers of such genes could potentially provide useful tools for targeting gene expression to specific KC subtypes (in combination with inter-sectional strategies using a general pan-KC-specific Split-GAL4) to visualize their morphology and to test whether they may have specific roles in learning and memory.

### Hemocyte, ring gland and glial clusters and their differentially expressed genes

Three major non-neuronal cell types were recovered from our dissociated nervous system samples: hemocytes, ring gland cells, and glia (Fig. 1b and e).

Three distinct clusters of hemocytes all shared *SPARC* expression (Fig. 1c and e) but showed cluster-specific gene expression (see Supplementary Fig. 19 and Supplementary Spreadsheet 2 for genes differentially expressed in each hemocyte cluster) [40].

The ring gland is a major output system of the brain in addition to descending neurons that control locomotion. The ring gland integrates input from a range of distinct neuronal pathways and secretes hormones and neuropeptides into the hemolymph [76]. These hormones can have wide-ranging effects on physiological processes and behaviour. We identified one cluster containing ring gland cells (cl. 66) and more than 180 genes whose expression was more than two-fold enriched in this cluster (Fig. 1b and e, Supplementary Fig. 20 and Supplementary Spreadsheet 2). Some of these were highly selectively expressed in the ring gland, including a number of CGs with unknown function, such as CG4408, CG5773, CG2991, CG44325, CG43886, CG43156, CG4456, CG6310. These provide promising candidates for genes involved in hormone synthesis and secretion.

Glial cells are non-neuronal components of the nervous system essential for its function and account for ca. 10% of its cells in *Drosophila*. 5 distinct subtypes of glia have been described in the *Drosophila* CNS: astrocyte-like, ensheathing, cortex and two kinds of surface glia (subperineural and perineural, Yildirim et al. 2018).

We identified a total of 8 distinct clusters of glial cells, based on their differential expression of known glial markers, such as *fabp*, *repo*, *tramtrack* etc. (Fig. 1b-c, e and Supplementary Spreadsheet 2) [37, 77–79]. We were able to further identify each individual glial cluster based on the expression of known markers for specific glial subtypes (Fig. 4a-e, Supplementary Fig. 21, Supplementary Spreadsheets 2 and 16). Two clusters (50 and 58) were enriched in astrocyte-like glial markers, *Gat*, *alrm* and G*s2* [80–82]. One of them (cl. 50) was also enriched in *ebony* and *Eaat1* (Fig. 4b) [83, 84]. Two clusters were enriched in known markers for ensheathing glia, although the markers segregated to distinct clusters (Fig. 4c). Thus, cluster 38 was highly enriched in *TRAF4*, while cluster 35 was highly enriched in *ClC-a*, *axotactin* and *CG9657* [85–87]. Three clusters (26, 27 and 52) were enriched in markers for cortex glia, such as wrapper, zydeco, hoe1 (Fig. 4d) [88, 89]. One cluster (55) was enriched in markers for subperineural glia, moody, MDR65 and Swim (Fig. 4e) [90–92].

We found numerous genes whose expression was highly enriched in each of these glial clusters (Fig. 4b-e, Supplementary Fig. 21 and Supplementary Spreadsheets 2 and 16). For example, a number of genes involved in nutrient transport (*Slc45-1*, *ZnT77C*, *Oatp58Dc*) and metabolism (*Dci*, *ACC*) were selectively enriched in cortex glia consistent with their proposed role in efficient transfer of nutrients from the haemolymph to neuronal cell bodies [37, 93–97]. Additionally, many genes with previously unknown functions were selectively enriched in specific glial clusters. For example, *CG40470*, *CG9451*, *CG43448*, *CG7997*, *CG11594* were selectively enriched only in cortical glial clusters; *CG9394*, *CG34367*, *CG8568* in astrocyte-like glia; *CG7860* in ensheathing glia; *CG10702*, *CG14855*, *CG1299* in subperineural glia. These genes could contribute to the function of specific glial classes.

Prior studies have reported a general larval glial transcriptome [21, 80], and transcriptomes of adult surface and astrocyte-like glia [37, 98, 99]. Here we extend the glial transcriptome to additional glial subtypes, ensheathing and cortex glia. These genes provide promising candidates further dissecting the functional roles of distinct glial subtypes.

### Differentially expressed genes in immature adult neurons

The larval nervous system also contains NPs and developing adult neurons. From early 2^nd^ instar, and in some cases earlier, NPs start producing developing adult neurons that grow their principal neurites and then stall, waiting to complete their projections during pupation [32, 33]).

Recent work in a 16 h.-old first instar brain showed that adult developing neurons express *headcase* (*hdc*) and cibulot (*cib*) [21]. We identified 13 distinct clusters of developing adult neurons that expressed these markers (Fig. 1b-d and 5a,c).

We also compared the numbers of developing adult neurons at distinct stages: 1 h., 24 h. and 48 h. From stage-specific atlases of the nervous systems (Fig. 6a) we observed changes in cellular composition of the nervous system during development. As expected, at 1 h, the nervous system primarily consists of mature larval neurons (Fig. 6a). As development proceeds, the proportion of *headcase*-expressing, developing adult neurons greatly expands (Fig. 6a, Supplementary Spreadsheet 17).

We find many transcriptional regulators that were highly enriched either in specific, or in all immature neuron clusters that could potentially regulate the differentiation of specific adult neuron types. Examples include E(spl)m6-BFM, pdm3, bric a brac 2 (bab2), ara, caup, acj6, mirr, salm, Erect wing (ewg) and others [100–107], as well as several predicted transcription factors with unknown functions (e.g., CG9650, CG32532, CG11294, Fig. 5c and Supplementary Spreadsheets 2 and 8).

Numerous cell surface molecules involved in axon guidance, dendritic morphogenesis were also highly enriched in these clusters, including the actin-binding protein *singed* (*sn*), robo2, fz2 and others (Fig. 5c and Supplementary Spreadsheets 2 and 8) [108–110]. These genes could potentially regulate the growth and targeting of the developing adult neurons.

Additionally, we found many genes with previously unknown function selectively enriched in these clusters, some of which were selectively enriched only in specific clusters (Supplementary Spreadsheets 2 and 8, Supplementary Fig. 22). Examples include CG12643, CG11030, CG5151, CG11294 (only in cl. 33), CG9016 (only in cl. 34) and others. Such genes could potentially regulate the differentiation of specific adult neuron types and provide promising candidates for future follow up studies.

### Differentially expressed genes in neuronal precursors

Neural progenitor cells include neuroblasts (NBs), intermediate neural progenitors (INPs), and ganglion mother cells (GMCs) [35]. NBs divide asymmetrically to produce progeny. Type I NBs divide into one NB and one GMC. Type II NBs divide into one neuroblast and one intermediate neural progenitor (INP) which then itself divides into a GMC [35]. Each GMC then divides terminally to form two neurons or one neuron and one glial cell.

We identified 6 distinct clusters that contained neural progenitor cells (collectively identified based on the expression of Notch (Figs. 1c-d and 5a,d, Supplementary Fig. 23 and Supplementary Spreadsheets 2 and 7). Clusters 40 and 41 highly differentially expressed known Type I NB markers (such as miranda, string, Cyclin E (Fig. 5d) but not Type II NB or INP markers (Supplementary Spreadsheets 2 and 7) [111–113]. Cluster 15 selectively differentially expressed known Type II NB markers (pointed and tailless, Fig. 5d) [114, 115]. However, this cluster also contained a large number of Notch expressing cells that did not express these markers. Differential marker expression suggests that at least some of these cells are optic-lobe neuroepithelia (Avalos, C.B. et al. 2019), as evidenced by their differential expression of Ocho, Bearded (Brd), Twin of m4 (Tom) and several members of the enhancer of split family (E(spl) (Supplementary Spreadsheet 2 and 7). Cluster 11 differentially expressed known INP markers (earmuff and hamlet, Fig. 5d and Supplementary Spreadsheets 2 and 7) [116, 117]. Cluster 2 differentially expressed known GMC markers (target of poxn, dacapo, Phaser, Fig. 5d) [118, 119]. Cluster 42 showed a different composition from all other neuronal precursors and was spatially segregated in the UMAP projection. Differentially expressed genes enriched in this cluster are associated with epithelial processes and trachea development [120–123]. Examples of such genes are piopio (pio), expansion (exp), papillote (pot) or dumpy (dpy) (Supplementary Spreadsheet 2 and 7).

We also confirmed this identification by performing a pseudotime analysis in Monocle3 [124–126]. For this purpose, we combined all NPCs and developing adult neurons and anchored the analysis to Type I (cluster 41) and Type II (cluster 15) NBs present at 1 h. This aligned the NPCs along a temporal axis where NB clusters were at the start GMC and INP clusters were in the middle and developing neuron clusters were at the end (Fig. 5b).

We identified a number of genes differentially expressed in each progenitor cluster (Fig. 5d, Supplementary Fig. 22 and Supplementary Spreadsheets 2 and 7). These genes could play roles in specifying distinct types of NPs and regulating their patterns of division.

### Developmental profile of gene expression across distinct life stages

Our datasets collected from three distinct life stages enables comparisons of gene expression between stages in each of the CNS clusters (Fig. 6a and Supplementary Spreadsheet 17). We wondered whether there would be any differences in gene expression between a 48-h. old larva and a 24-h. old larva in the distinct types of functional larval neuron classes and glia. In a companion study in the same issue, we explore in more detail the genes that are differentially expressed at different stages in NPs and immature adult neurons (Dillon et al. submitted). Such genes provide interesting candidates for specifying the temporal identities of NPCs and their progeny neurons.

A number of genes were more than twofold enriched in neurons at 48 h. compared to 24 h. (Fig. 6b and Supplementary Spreadsheet 17). Most of these genes were enriched at the later life stage in most or all neuron classes. Some were enriched at 48 h. only in specific neuron classes. For example, sNPF was selectively enriched in KCs [127]. CG9336 was selectively enriched in peptidergic and octopaminergic neurons.

Similarly, most genes that were differentially expressed in glia at 48 h were differentially expressed in all glial classes, while a few were selectively enriched only in a specific glial class hrs. (Fig. 6c and Supplementary Spreadsheet 17). For example, CG14687 was selectively enriched at 48 h compared to 24 h in astrocyte glia, while CG40470 and Slc45-1 in cortex glia.

It will be interesting to explore in future studies what roles these genes might play in functional larval neurons and glia at later stages of larval life.

### Validating transcriptomic predictions

Our Atlas reveals numerous genes that are differentially expressed in specific classes of *Drosophila* neurons and at specific stages of development. We wanted to validate using RNA-FISH the expression pattern of at least one of the genes newly discovered here to be differentially expressed in a specific cell type [23, 128]. For this purpose, our aim was to use a cell type containing relatively few cells and look for co-expression of a previously known marker with the newly discovered one.

**Fig. 7:**
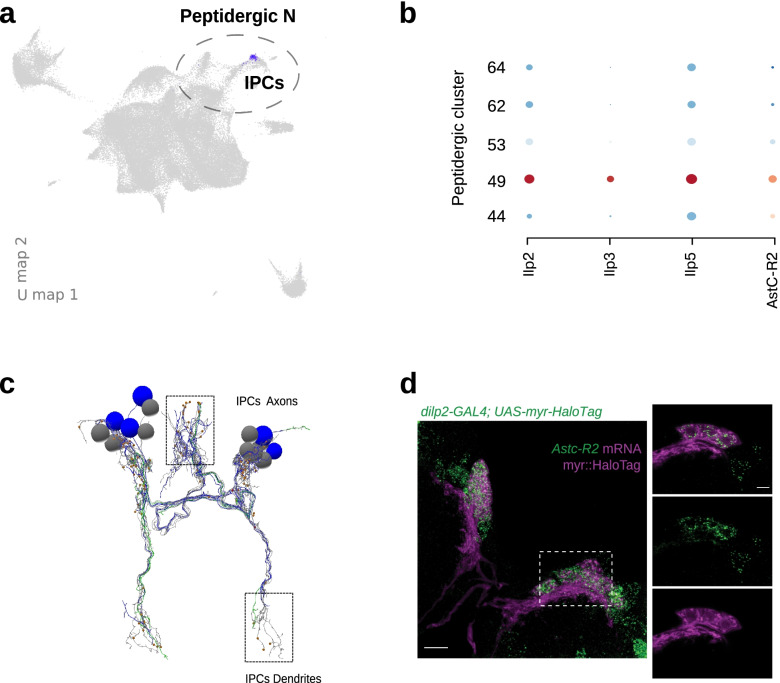
Validation of AstC-R2 expression in insulin producing cells. Experimental validation of the presence of an undecribed isoform of allostatin receptor (AstC-R2) in a well characterized cell type, insulin producing cells, for which GAL4 driver lines are available enabling fluorescence co-localization of the receptor in the aforementioned neurons. **a** Featureplot of mature neurons. Among peptidergic clusters (circled) cells with robust expression of insuline like peptides Ilp2, Ilp3 and Ilp5 are colored in purple. IPCs: Insulin producing cells. **b** Maximum-intensity projection of the confocal stack of a brain in which the IPCs are labeled with a fluorescent HaloTag ligand (Magenta) and AstC-R2 mRNA is co-detected by FISH (green), bar 10 µm. Dashed lines outline area where the single z plane is shown on the left panels, bar 5 µm. **c** Dotplot showing the enriched expression of Ilp2, Ilp3, Ilp5 and AstC-R2 in peptidergic cluster 49 compared to the remaining peptidergic clusters (numering as in Fig. 1b). **d** Anatomy of insulin-producing cells (IPCs). The IPCs are a group of 7 bilaterally symmetrical neurons receiving input coveying the nutritional state of the animal through their dendrites (boxed, bottom). To respond, they release insulin-like peptides (Ilp2, Ilp3, Ilp5) through their axons (boxed, top), which synapse onto the ring gland, a major secretory organ responsible for homeostatic balance in Drosophila

We chose Insulin-producing cells (IPCs) as illustrative examples to validate scRNA-seq data (Fig. 7a-d). IPCs consist of just 14 neurons in the larval brain (Fig. 7c, [129]. These cells participate in circuits which monitor the nutritional status of the larva and function as the larval equivalent of the mammalian pancreas. If IPCs are ablated, larvae and adults are smaller and have a diabetic phenotype, including increased hemolymph trehalose and glucose levels [130]. IPCs secrete insulin-like peptides which regulate hemolymph sugar levels. One of our peptidergic clusters (49) was defined by strong differential expression of insulin-like peptides 2, 3 and 5 (*Ilp2, ilp3, ilp5*), which are canonical markers of IPCs (Fig. 7a-b) [131, 132]. We discovered multiple genes with a more than two-fold enrichment in this cluster, including some that were selectively differentially expressed only in that cluster and in a few other peptidergic clusters (Supplementary Fig. 12 and Supplementary Spreadsheet 18). One such gene was Allostatin C Receptor 2 (*AstC-R2*) that was not previously known to be expressed in this cell type (Fig. 7b) [24].

To validate the specificity of our scRNAseq approach for identifying AstC-R2 in IPC cells, we probed AstC-R2 mRNA in a HaloTag reporter line for the IPCs [133]. The overlap between the neurons containing the HaloTag and FISH signals confirmed the sequencing result (Fig. 7d). The colocalization of AstC-R2 with 14 IPCs suggests that all IPCs are regulated by AstC through AstC-R2. The discovery of regulation by AstC-R2 updates our model of the regulation of IPCs by adding an additional population of cells that are modulating IPC activity.

## Discussion

In this study we present the first full transcriptomic atlas of the entire central nervous system of *Drosophila* larva with single-cell resolution across multiple life stages. Unbiased clustering of more than 131,077 cells sequenced in this study based on their patterns of gene expression revealed 67 clusters of nervous system cells. We were able to identify the majority of the clusters based on known markers for distinct cell types. These included functional larval neurons, glial cells, neuronal precursors, and developing adult neurons, as well as ring gland and hemocyte clusters. For each cluster we identified large numbers of genes that were differentially expressed in that cluster compared to all other clusters. This dataset provides a valuable resource for studying genes involved in the development and function of the nervous system.

### Atlas of genes enriched in each neuron cluster provides a powerful resource for bridging the gap between genes, circuits and behaviour

For the most part, functional larval neurons segregated based on neurotransmitter identity. Thus, different clusters contained cholinergic, GABAergic, glutamatergic, octopaminergic, serotonergic, dopaminergic and peptidergic neurons. The neurons implicated in memory formation (MB KCs) and motor neurons (MNs), each formed separate clusters that were very different in terms of gene expression to all other functional larval neurons. Each of these major neuronal classes selectively expressed a specific known marker (e.g., genes involved in neurotransmitter synthesis or transport). Additionally, we identified numerous genes with previously unknown functions whose expression was highly enriched in each of these major classes, providing a rich set of candidate genes that could be required for proper function of these neuron classes (Supplementary Spreadsheet 5). Some of these were selectively expressed just in a single neurotransmitter class (Supplementary Spreadsheet 18), while others were expressed in specific combinations of classes.

We also searched for genes that were differentially expressed between individual clusters of the same neurotransmitter and found a number of such genes. Together with the selective pan-neurotransmitter markers, the markers that distinguish individual clusters of the same neurotransmitter could enable unique identification of that cluster.

At this stage we were unable to link the individual neuron clusters to specific morphologically characterized known neuron types. However, the combinations of genes that were differentially expressed in each cluster could provide a basis for determining the morphology, connectivity and functional roles of many of the clusters in the future. These markers could potentially enable selective targeting of gene expression to individual neuron clusters using intersectional techniques available in *Drosophila*. For example, knocking in Split-DBD (or AD) lines upstream of one marker gene and a Split-AD (or DBD) into the other could result in selective driver lines for single clusters [134–136]. Such lines would enable future anatomical and functional characterization of some of the neuronal clusters identified in this study. For example, selective expression of GFP would reveal their morphology. Furthermore, since most larval neurons can be uniquely identified based on their morphology, this would also reveal the connectivity patterns of neurons that have been reconstructed from electron microscopy.

### Gene expression atlas at distinct stages provides a resource for studying age-related changes in larval neurons and the specification of adult neurons

Our dataset also provides a resource for studying temporal changes in gene expression across larval life stages in specific types of larval neurons, glial cells, the ring gland, developing adult neurons and neural precursor cells. Genes that are differentially expressed in functional larval neurons at late stages of larval life could play roles in regulating larval molting and transitions between instar stages and in responding to hormones such as Ecdysone, juvenile hormone or prothoracicotropic hormone.

Since temporal cascades of transcription factor expression in neural precursors specify the fate of progeny neurons, the data generated in this study provides a rich resource for identifying novel factors that could be involved in neural fate specification and development. We pursue this question in more depth using this dataset in a companion study in the same issue.

## Conclusions

In summary, single-cell transcriptomic atlases are the missing piece required for the combined analysis of genes, circuits and behavior. By adding a transcriptomic atlas to the atlases of neuron connectivity, neuron activity and behavior, we have set the stage for a more complete understanding of the principles that underlie the complex interplay of genes, circuits, and behavior.

## Supplementary Information

**Additional file 1: Supplementary Spreadsheets and Figures.** All spreadsheets for marker genes contain the following columns**:** p-value (pval), average log2 fold-change (avg_log2FC), percent of cells in the cluster expressing the marker (pct.1), percent of cells outside the cluster expressing the marker (pct.2), the multiple test corrected p-value (p_val_adj), the cluster number (cluster), the gene name (gene), the flybase id (Fbgn_ID), gene long name (GeneName), datestamp of flybase snapshot inclusion (datestamp) and the Flybase gene snapshot for the gene in question, when available.(gene_snapshot_text). **Supplementary_spreadsheet_1_Time_and_tissue_breakdown.ods.** Spreadsheet detailing the number of cells per cluster and sample of origin, a stage by cell number breakdown and sequencing quality control metrics for each sequenced sample. **Supplementary_spreadsheet_2_Ncells_and_gene_markers_per_cluster.xlsx.** Spreadsheet containing one sheet per detected cluster with all the cluster defining markers resulting from running the FindAllMarkers algorithm as detailed in the methods. An additional sheet contains the number of cells per cluster. **Supplementary_spreadsheet_3_Ncells_and_gene_markers_per_cluster_and_stage.xlsx.** Spreadsheet containing one sheet per detected cluster with all the cluster defining markers at each stage, ie. 1h, 24h and 48h resulting from running the FindAllMarkers algorithm as detailed in the methods for the temporal analysis. An additional sheet contains the number of cells per cluster at each stage. **Supplementary_spreadsheet_4_Ncells_and_gene_markers_per_cluster_and_tissue.xlsx.** Spreadsheet containing one sheet per detected cluster with all the cluster defining markers for each tissue, ie. brain, CNS and VNC, resulting from running the FindAllMarkers algorithm as detailed in the methods for the temporal analysis. An additional sheet contains the number of cells per cluster detected in each tissue dissection. **Supplementary_spreadsheet_5_Differential_expression_cluster_mature_neuron_classes.ods.** Spreadsheet containing one sheet per mature neuron subtype and their markers resulting from running the FindAllMarkers algorithm as detailed in the methods but restricting it to mature cell-types only: Cholinergic, Gabaergic, Glutamatergic, Kenyon Cells, Motor, Monoaminergic and Peptidergic neurons. **Supplementary_spreadsheet_6_Differential_expression_cluster_big_classes.ods. **Spreadsheet containing one sheet per major cell-type class and their defining markers: Immature neurons, Cholinergic neurons, Neuroprecursor cells, Gabaergic neurons, Glutamatergic neurons, Kenyon cells, Unknown neurons, Motorneurons, Glia, Hemocytes, Epithelia/trachea, Monoaminergic neurons, Peptidergic neurons and Ring Gland. **Supplementary_spreadsheet_7_NPCs_markers_among.xlsx.** Spreadsheet containing one sheet per Neuroprecursor cluster and their markers resulting from running the FindAllMarkers algorithm as detailed in the methods but restricting it to Neuroprecursor clusters only. **Supplementary_spreadsheet_8_Immature_neuron_markers_among.xlsx.** Spreadsheet containing one sheet per Immature neuron cluster and their markers resulting from running the FindAllMarkers algorithm as detailed in the methods but restricting it to Immature neuron clusters only. **Supplementary_spreadsheet_9_Cholinergic_markers_among.xlsx.** Spreadsheet containing one sheet per Cholinergic cluster and their markers resulting from running the FindAllMarkers algorithm as detailed in the methods but restricting it to Cholinergic clusters only. **Supplementary_spreadsheet_10_Gabaergic_markers_among.xlsx. **Spreadsheet containing one sheet per Gabaergic cluster and their markers resulting from running the FindAllMarkers algorithm as detailed in the methods but restricting it to Gabaergic clusters only. **Supplementary_spreadsheet_11_Glutamatergic_markers_among.xlsx.** Spreadsheet containing one sheet per Glutamatergic cluster and their markers resulting from running the FindAllMarkers algorithm as detailed in the methods but restricting it to Glutamatergic clusters only. **Supplementary_spreadsheet_12_Octopaminergic_markers_among.xlsx. **Spreadsheet containing one sheet per Octopaminergic cluster and their markers resulting from running the FindAllMarkers algorithm as detailed in the methods but restricting it to Octopaminergic clusters only. **Supplementary_spreadsheet_13_Serotoninergic_markers_among.xlsx. **Spreadsheet containing one sheet per Serotoninergic cluster and their markers resulting from running the FindAllMarkers algorithm as detailed in the methods but restricting it to Serotoninergic clusters only. **Supplementary_spreadsheet_14_Peptidergic_markers_among.xlsx. **Spreadsheet containing one sheet per Peptidergic cluster and their markers resulting from running the FindAllMarkers algorithm as detailed in the methods but restricting it to Peptidergic clusters only. **Supplementary_spreadsheet_15_Kenyon-Cells_markers_among.xlsx.** Spreadsheet containing one sheet per Kenyon cells cluster and their markers resulting from running the FindAllMarkers algorithm as detailed in the methods but restricting it to Kenyon cells clusters only. **Supplementary_spreadsheet_16_Glia_markers_among.xlsx.** Spreadsheet containing one sheet per Glia cluster and their markers resulting from running the FindAllMarkers algorithm as detailed in the methods but restricting it to Glia clusters only. **Supplementary_spreadsheet_17_Enriched_markers_per_cluster_48_vs_24h.xlsx.** Spreadsheet containing one sheet per big cell-type class with all the markers enriched at 48h vs 24h resulting from running the FindAllMarkers algorithm as detailed in the methods for the temporal analysis but restricting it to 48 vs 24h. **Supplementary_spreadsheet_18_selective_one_per_class_075-19.xlsx.** Spreadsheet containing one sheet per cluster with all markers selective for that cluster when imposing a cut-off of log2 fold-change greater than 0.75 and the requirement of being detected in more than 19% of cells. **Supplementary_spreadsheet_19_Identity_markers_and_refs.ods.** Spreadsheet containing the list of all markers used to identify cell classes together with literature references. **Supplementary_spreadsheet_20_Brain_only_atlas_markers.xlsx.** Spreadsheet containing one sheet per cluster with all markers selective for that cluster when imposing a cut-off of log2 fold-change greater than 0.75 and the requirement of being detected in more than 19% of cells. In the Brain samples and the VNC samples it can be seen that there is a drastic increase of immature neurons relative to mature neurons from 24 hrs to 48 hrs. In the Brain samples, at 24 hrs, the ratio of immature (4885) to mature neurons (8536) is 0.57; at 48 hrs the ratio of immature (12092) to mature neurons (9758) is 1.23 (2.2-fold increase). **Supplementary_spreadsheet_21_VNC_only_atlas_markers.xlsx.** Spreadsheet containing one sheet per cluster with all markers selective for that cluster when imposing a cut-off of log2 fold-change greater than 0.75 and the requirement of being detected in more than 19% of cells. In the Brain samples and the VNC samples it can be seen that there is a drastic increase of immature neurons relative to mature neurons from 24 hrs to 48 hrs. In the VNC samples, at 24 hrs, the ratio of immature (3146) to mature neurons (4885) is 0.64; At 48 hrs the ratio of mature (2173) to immature (3513) is 1.61 (2.5-fold increase). **Supplementary_Figure_1_UMAP_plot_per_tissue.pdf. **UMAP representation of the CNS cell type diversity discovered after reciprocal-PCA integration, dimensionality reduction and unsupervised clustering with Seurat and split by tissue of origin. In this 2D representation each dot represents a cell and their distribution in space is a function of their similarity in gene expression profile. Each cluster is color and number coded as depicted in the accompanying legend. **Supplementary_Figure_2_Brain_independent_analysis.pdf. **UMAP dimensional reduction plot with the annotated clustering resulting from the analysis of VNC samples only at 24 and 48h. **Supplementary_Figure_3_VNC_independent_analysis.pdf. **UMAP dimensional reduction plot with the annotated clustering resulting from the analysis of Brain samples only at 24 and 48h. **Supplementary_Figure_4_endogenous-nSyb-feature_plot.pdf. **Feature plot comparing the expression distribution of endogenous and UAS-GAL4 amplified expression of nSyb. **Supplementary_Figure_5_feature_plot_nSyb_Repo_Notch.pdf. **UMAP dimensional reduction showing the expression distribution of endogenous nSyb, repo and Notch. In this 2D representation each dot represents a cell and their distribution in space is a function of their similarity in gene expression profile. Color represents the expression of the gene for that particular cell. In each dotplot, the centered mean expression of a gene for each class is calculated and given a color ranging from blue (lowest expression) to red (highest expression), with white corresponding to 0. In this fashion different genes can be compared by their relative expression in the classes depicted irrespective of their absolute expression levels. The diameter of each dot is proportional to the number of cells expressing that gene in the class. **Supplementary_Figure_6_cholinergic_markers_dotplot.pdf. **Dotplot depicting Cholinergic markers showing an average log2 fold-change greater than one compared to the other clusters and present in at least more than 19% of the cells of the cluster. **Supplementary_Figure_7_glutamatergic_markers_dotplot.pdf. **Dotplot depicting Glutamatergic markers showing an average log2 fold-change greater than one compared to the other clusters and present in at least more than 19% of the cells of the cluster. **Supplementary_Figure_8_gabaergic_markers_dotplot.pdf.** Dotplot depicting Gabaergic markers showing an average log2 fold-change greater than one compared to the other clusters and present in at least more than 19% of the cells of the cluster. **Supplementary_Figure_9_octopaminergic_markers_dotplot.pdf. **Dotplot depicting Octopaminergic markers showing an average log2 fold-change greater than one compared to the other clusters and present in at least more than 19% of the cells of the cluster. **Supplementary_Figure_10_serotoninergic_markers_dotplot.pdf. **Dotplot depicting Serotoninergic markers showing an average log2 fold-change greater than one compared to the other clusters and present in at least more than 19% of the cells of the cluster. **Supplementary_Figure_11_dopaminergic_markers_dotplot.pdf. **Dotplot depicting Dopaminergic markers showing an average log2 fold-change greater than one compared to the other clusters and present in at least more than 19% of the cells of the cluster. **Supplementary_Figure_12_peptidergic_markers_dotplot.pdf. **Dotplot depicting Peptidergic markers showing an average log2 fold-change greater than one compared to the other clusters and present in at least more than 19% of the cells of the cluster. **Supplementary_Figure_13_Cholinergic_among_markers_dotplot.pdf. **Dotplot depicting Cholinergic markers showing an average log2 fold-change greater than one compared to the other Cholinergic clusters and present in at least more than 19% of the cells of the cluster. **Supplementary_Figure_14_Glutamatergic_among_markers_dotplot.pdf. **Dotplot depicting Glutamatergic markers showing an average log2 fold-change greater than one compared to the other Glutamatergic clusters and present in at least more than 19% of the cells of the cluster. **Supplementary_Figure_15_cotransmitter_upset_number.pdf. **Histogram with numbers and percent of cells expressing combinations of one, two, three and four fast acting neurotrasmitters compared to single neurotransmitter expressing ones. **Supplementary_Figure_16_Gabaergic_among_markers_dotplot.pdf. **Dotplot depicting Gabaergic markers showing an average log2 fold-change greater than one compared to the other Gabaergic clusters and present in at least more than 19% of the cells of the cluster. **Supplementary_Figure_17_Octopaminergic_among_markers_dotplot.pdf. **Dotplot depicting Octopaminergic markers showing an average log2 fold-change greater than one compared to the other Octopaminergic clusters and present in at least more than 19% of the cells of the cluster. **Supplementary_Figure_18_Serotoninergic_among_markers_dotplot.pdf. **Dotplot depicting Serotoninergic markers showing an average log2 fold-change greater than one compared to the other Serotoninergic clusters and present in at least more than 19% of the cells of the cluster. **Supplementary_Figure_19_hemocytes_markers_dotplot.pdf. **Dotplot depicting Hemocyte markers showing an average log2 fold-change greater than one compared to the other clusters and present in at least more than 19% of the cells of the cluster. **Supplementary_Figure_20_ring-gland_markers_dotplot.pdf. **Dotplot depicting Ring gland markers showing an average log2 fold-change greater than one compared to the other clusters and present in at least more than 19% of the cells of the cluster. **Supplementary_Figure_21_Glia_among_markers_dotplot.pdf. **Dotplot depicting Glia markers showing an average log2 fold-change greater than one compared to the other Glia clusters and present in at least more than 19% of the cells of the cluster. **Supplementary_Figure_22_Immature_among_markers_dotplot.pdf. **Dotplot depicting Immature neuron markers showing an average log2 fold-change greater than one compared to the other Immature clusters and present in at least more than 19% of the cells of the cluster. **Supplementary_Figure_23_Npcs_among_markers_dotplot.pdf. **Dotplot depicting Immature neuron markers showing an average log2 fold-change greater than one compared to the other Immature clusters and present in at least more than 19% of the cells of the cluster.

## Data Availability

Raw sequencing data was deposited at GEO (accession number: GSE135810) and can be accessed at: https://www.ncbi.nlm.nih.gov/geo/query/acc.cgi?acc=GSE135810 All the code to carry the analysis and the environment for reproducible pipeline execution can be found at https://github.com/histonemark/Brainseq_code

## Abbreviations

scRNAseq: High-throughput single-cell RNA sequencing
RNA-FISH: RNA fluorescent in situ hybridization
ALH: After larval hatching
CNS: Central nervous system
VNC: Ventral nerve cord
UMAP: Uniform Manifold Approximation and Projection
NPs: Neural precursor cells
IANs: Immature adult neurons
G: Glial cells
RG: Ring gland
Hm: Hemocytes
KCs: Kenyon cells
MB: Mushroom body
MLNs: Mature larval neuron
nSyb: N-synaptobrevin
N: Notch
hdc: Headcase
cib: Cibulot
UNs: Unknown neurons
Ch: Cholinergic neurons
Glu: Glutamatergic neurons
MNs: Motor neurons
MA: Monoaminergic neurons
OA: Octopaminergic neurons
Ser: Serotonergic neurons
DA: Dopaminergic neurons
Pt: Peptidergic neurons
spab: Space blanket
grh: Grainy head
qm: Quemao
PN: Projection neuron
sn: Singed
NBs: Neuroblasts
INPs: Intermediate neural progenitors
GMCs: Ganglion mother cells
INP: Intermediate neural progenitor
IPCs: Insulin-producing cells
AstC-R2: Allostatin C Receptor 2
