# Correction: A single-cell transcriptomic atlas of complete insect nervous systems across multiple life stages

**DOI:** 10.1186/s13064-022-00167-3

**Published:** 2022-10-26

**Authors:** Marc Corrales, Benjamin T. Cocanougher, Andrea B. Kohn, Jason D. Wittenbach, Xi S. Long, Andrew Lemire, Albert Cardona, Robert H. Singer, Leonid L. Moroz, Marta Zlatic

**Affiliations:** 1 Howard Hughes Medical Institute Janelia Research Campus, Ashburn, VA USA; 2 Department of Physiology, Development, and Neuroscience, Cambridge University, Cambridge, UK; 3 Department of Zoology, Cambridge University, Cambridge, UK; 4 Department of Neuroscience and Whitney Laboratory for Marine Biosciences, University of Florida, Gainesville/St. Augustine, FL 32080 USA; 5 MRC Laboratory of Molecular Biology, Cambridge Biomedical Campus, Francis Crick Avenue, Cambridge, UK; 6 Department of Anatomy and Structural Biology, Albert Einstein College of Medicine, Bronx, NY USA

**Correction: Neural Dev 17, 8 (2022)**

10.1186/s13064-022-00164-6

Following publication of the original article [1], the authors identified an error in Fig. 6. The correct figure is shown on the following page.

**Fig. 6:**
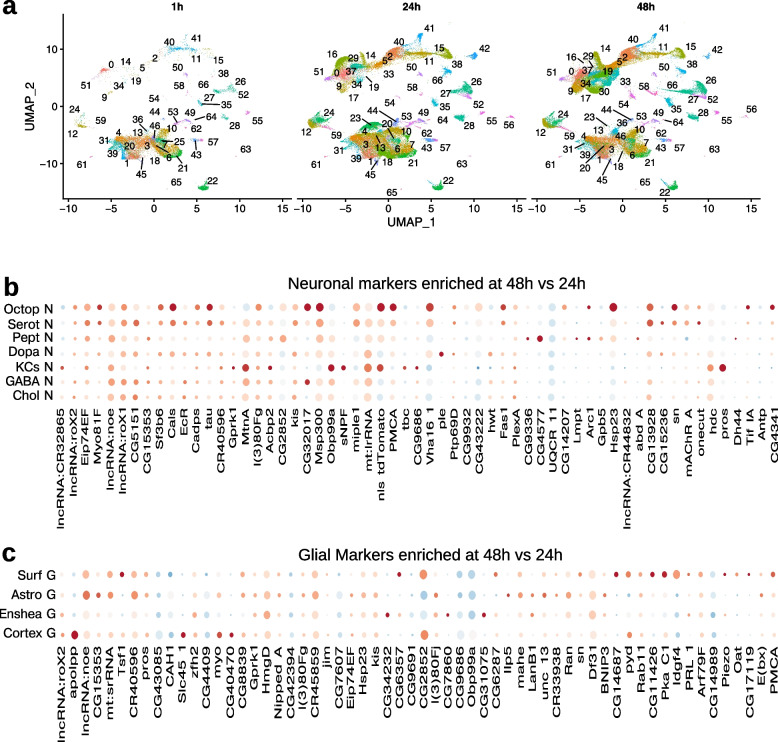
Temporal changes in marker composition. The Seurat algorithm of reciprocal-PCA allows to find matched populations of cells across our differently aged samples enabling the differential gene expression analysis of a given cell type at 1, 24 and 48 h age. **a** Same UMAP plot as in Fig. 1b, but separated per stage, showing the matched clusters when present. The numbering of the clusters is the same as in Fig. 1b. 1 h, 24 h, 48 h, refer to age in hours post hatching and prior to dissection. **b** Dotplot depicting the topmost temporally varible genes for mature neurons. Chol N: Cholinergic neurons, GABA N: Gabaergic neurons, KCs: Kenyon cell neurons, Dopa N: Dopaminergic neurons, Pept N: Peptidergic neurons, Serot N: Serotoninergic neurons, Octop N: Octopaminergic neurons. **c** Dotplot depicting the topmost temporally varible genes for glial subclasses. Cortex G: Cortex glia, Enshea G: Ensheathing glia, Astro G: Astrocyte glia, Surf G: Surface glia

The original article has been corrected.
