# Supplementary material for: A single-cell transcriptomic atlas of complete insect nervous systems across multiple life stages
Source: Neural Dev. 2022 Aug 24;17:8. doi: 10.1186/s13064-022-00164-6 (PMC9404646; doi:10.1186/s13064-022-00164-6)
Supplement: Supplementary file 1 — Additional file 1: Supplementary Spreadsheets and Figures. All spreadsheets for marker genes contain the following columns: p-value (pval), average log2 fold-change (avg_log2FC), percent of cells in the cluster expressing the marker (pct.1), percent of cells outside the cluster expressing the marker (pct.2), the multiple test corrected p-value (p_val_adj), the cluster number (cluster), the gene name (gene), the flybase id (Fbgn_ID), gene long name (GeneName), datestamp of flybase snapshot inclusion (datestamp) and the Flybase gene snapshot for the gene in question, when available.(gene_snapshot_text). Supplementary_spreadsheet_1_Time_and_tissue_breakdown.ods. Spreadsheet detailing the number of cells per cluster and sample of origin, a stage by cell number breakdown and sequencing quality control metrics for each sequenced sample. Supplementary_spreadsheet_2_Ncells_and_gene_markers_per_cluster.xlsx. Spreadsheet containing one sheet per detected cluster with all the cluster defining markers resulting from running the FindAllMarkers algorithm as detailed in the methods. An additional sheet contains the number of cells per cluster. Supplementary_spreadsheet_3_Ncells_and_gene_markers_per_cluster_and_stage.xlsx. Spreadsheet containing one sheet per detected cluster with all the cluster defining markers at each stage, ie. 1h, 24h and 48h resulting from running the FindAllMarkers algorithm as detailed in the methods for the temporal analysis. An additional sheet contains the number of cells per cluster at each stage. Supplementary_spreadsheet_4_Ncells_and_gene_markers_per_cluster_and_tissue.xlsx. Spreadsheet containing one sheet per detected cluster with all the cluster defining markers for each tissue, ie. brain, CNS and VNC, resulting from running the FindAllMarkers algorithm as detailed in the methods for the temporal analysis. An additional sheet contains the number of cells per cluster detected in each tissue dissection. Supplementary_spreadsheet_5_Differential_expres [file 13064_2022_164_MOESM1_ESM.zip › Supplementary/Supplementary_Spreadsheets_and_table/Supplementary_Table_Key-resources .docx]

| REAGENT or RESOURCE | SOURCE | IDENTIFIER |
| --- | --- | --- |
| Antibodies | | |
| FISH probes | Biosearch Technologies |  |
| RNase-free 1x PBS | Fisher Scientific | BP2438-4 |
| Acetic Acid, Glacial (Certified ACS), Fisher Chemical | Fisher Scientific | A38S-500 |
| Sodium borohydride, 99%, VenPure™ SF powder | Acros Organics | AC448481000 |
| SSC (20X) | Fisher | AM9763 |
| Hi-Di formamide | Applied Biosystems | 4311320 |
| Denhardt's solution (50X) | Alfa Aesar | AAJ63135AD |
| tRNA from baker's yeast | Roche | 10109495001 |
| UltraPure™ Salmon Sperm DNA Solution | Fisher Scientific | 15632011 |
| SDS, 10% | Corning | 46-040-CI |
| Deionizedformamide | Ambion | AM9342 |
| Critical Commercial Assays | | |
| Chromium Single Cell 30 Library & Gel Bead Kit v2 | 10x Genomics | PN-120237 |
| Chromium Single Cell A Chip Kit | 10x Genomics | PN-120236 |
| Chromium i7 Multiplex Kit | 10x Genomics | PN-120262 |
| Deposited Data | | |
| Raw and analyzed scRNAseq data | This paper | GEO: GSE135810 |
| Experimental Models: Organisms/Strains | | |
| *D. melanogaster*: w[1118]; P{y[+t7.7] w[+mC]=GMR57C10-GAL4}attP2 | Bloomington *Drosophila* Stock Center | RRID:BDSC_39171 |
| *D. melanogaster*: pJFRC29-10XUAS-IVS-myr::GFP-p10 in attP40; pJFRC105-10XUAS-IVS-nlstdTomato in VK00040 | Jack Etheredge; Etheredge, 2017 | N/A |
| *D. melanogaster*: w[1118]; P{y[+t7.7] w[+mC]=GMR72F11-GAL4}attP2 | Bloomington *Drosophila* Stock Center | RRID:BDSC_39786 |
| *D. melanogaster*: P{GawB}Tab2[201Y] | Bloomington *Drosophila* Stock Center | RRID:BDSC_4440 |
| *D. melanogaster*: w[1118] P{y[+t7.7] w[+mC]=20XUAS-IVS-CsChrimson.mVenus}attP18 | Bloomington *Drosophila* Stock Center | RRID: BDSC_55134 |
| Software | | |
| bcl2fastq | Ilumina | https://support.illumina.com/sequencing/ sequencing_software/bcl2fastq-  conversion-software.html; RRID: SCR_015058 |
| Cell Ranger | 10x Genomics | <https://support.10xgenomics.com/single-cell-gene-expression/software/overview/welcome> |
| R | R Core Team (2020). R: A language and environment for statistical computing. R Foundation for  Statistical Computing, Vienna, Austria. | https://www.R-project.org/ |
| Guix |  | https://guix.gnu.org/ |
| Seurat | Satija et al., 2015 | <https://satijalab.org/seurat/>; RRID: SCR_007322 |
| Monocle3 | Cao et al., 2019 | <https://cole-trapnell-lab.github.io/monocle3/> |
| Fiji | [PMID:22743772](http://www.ncbi.nlm.nih.gov/pubmed/22743772) | [http://fiji.sc](http://fiji.sc/); RRID:SCR_002285 |
| Other | | |
| Analysis pipeline | This paper | https://github.com/histonemark/Brainseq_code |
