# Supplementary material for: A single-cell transcriptomic atlas of complete insect nervous systems across multiple life stages
Source: Neural Dev. 2022 Aug 24;17:8. doi: 10.1186/s13064-022-00164-6 (PMC9404646; doi:10.1186/s13064-022-00164-6)
Supplement: Supplementary file 1 — Additional file 1: Supplementary Spreadsheets and Figures. All spreadsheets for marker genes contain the following columns: p-value (pval), average log2 fold-change (avg_log2FC), percent of cells in the cluster expressing the marker (pct.1), percent of cells outside the cluster expressing the marker (pct.2), the multiple test corrected p-value (p_val_adj), the cluster number (cluster), the gene name (gene), the flybase id (Fbgn_ID), gene long name (GeneName), datestamp of flybase snapshot inclusion (datestamp) and the Flybase gene snapshot for the gene in question, when available.(gene_snapshot_text). Supplementary_spreadsheet_1_Time_and_tissue_breakdown.ods. Spreadsheet detailing the number of cells per cluster and sample of origin, a stage by cell number breakdown and sequencing quality control metrics for each sequenced sample. Supplementary_spreadsheet_2_Ncells_and_gene_markers_per_cluster.xlsx. Spreadsheet containing one sheet per detected cluster with all the cluster defining markers resulting from running the FindAllMarkers algorithm as detailed in the methods. An additional sheet contains the number of cells per cluster. Supplementary_spreadsheet_3_Ncells_and_gene_markers_per_cluster_and_stage.xlsx. Spreadsheet containing one sheet per detected cluster with all the cluster defining markers at each stage, ie. 1h, 24h and 48h resulting from running the FindAllMarkers algorithm as detailed in the methods for the temporal analysis. An additional sheet contains the number of cells per cluster at each stage. Supplementary_spreadsheet_4_Ncells_and_gene_markers_per_cluster_and_tissue.xlsx. Spreadsheet containing one sheet per detected cluster with all the cluster defining markers for each tissue, ie. brain, CNS and VNC, resulting from running the FindAllMarkers algorithm as detailed in the methods for the temporal analysis. An additional sheet contains the number of cells per cluster detected in each tissue dissection. Supplementary_spreadsheet_5_Differential_expres [file Supplementary_Figure_9_octopaminergic_markers_dotplot.pdf › Supplementary/Individual_supplementary_figures/Supplementary_Figure_9_octopaminergic_markers_dotplot.pdf]

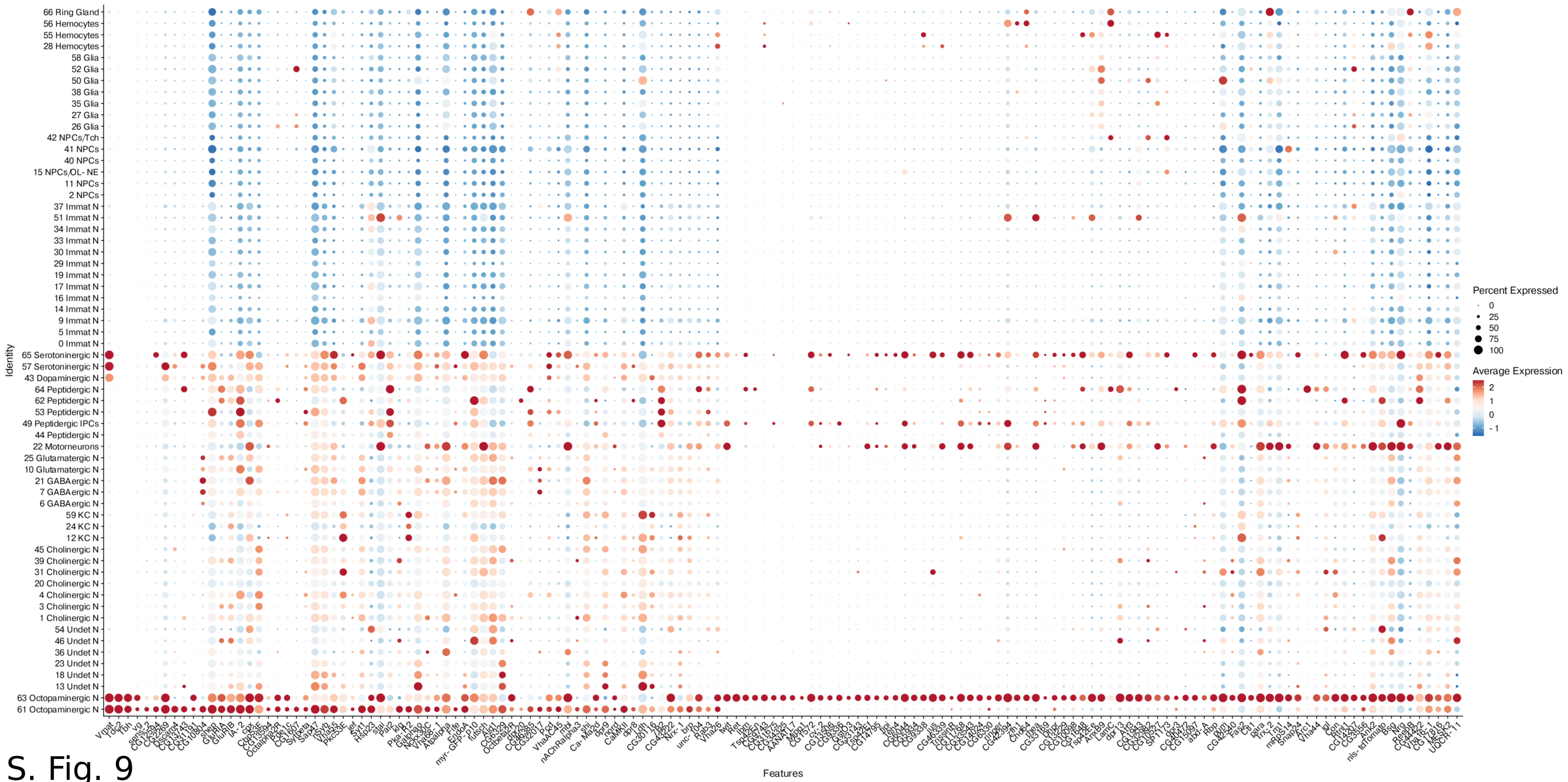

S. Fig. 9
